# Differences in glycolytic metabolism between tissue-resident alveolar macrophages and recruited lung macrophages

**DOI:** 10.3389/fimmu.2025.1535796

**Published:** 2025-02-28

**Authors:** Parker S. Woods, Gökhan M. Mutlu

**Affiliations:** Department of Medicine, Section of Pulmonary and Critical Care Medicine, The University of Chicago, Chicago, IL, United States

**Keywords:** alveolar macrophage, monocyte-derived alveolar macrophage, interstitial macrophage, bone marrow-derived macrophage, metabolism, glycolysis, bioenergetics, cytokines

## Abstract

Immunometabolism has emerged as a key area of focus in immunology and has the potential to lead to new treatments for immune-related diseases. It is well-established that glycolytic metabolism is essential for adaptation to hypoxia and for macrophage inflammatory function. Macrophages have been shown to upregulate their glycolytic metabolism in response to pathogens and pathogen-associated molecular patterns such as LPS. As a direct link to the external environment, the lungs’ distinctive nutrient composition and multiple macrophage subtypes provide a unique opportunity to study macrophage metabolism. This review aims to highlight how the steady-state airway and severely inflamed airway offer divergent environments for macrophage glycolytic metabolism. We describe the differences in glycolytic metabolism between tissue-resident alveolar macrophages, and other lung macrophages at steady-state and during inflammation/injury. We also provide an overview of experimental guidelines on how to assess metabolism at the cellular level using Seahorse-based bioenergetic analysis including a review of pharmacologic agents used to inhibit or activate glycolysis.

## 1 Introduction

It is well-established that glycolytic metabolism is essential for adaptation to hypoxia and for macrophage inflammatory function. Macrophages have been shown to upregulate their glycolytic metabolism in response to pathogens and pathogen-associated molecular patterns such as LPS. As a direct link to the external environment, the lungs’ distinctive nutrient composition and multiple macrophage subtypes provide a unique opportunity to study macrophage metabolism. This review aims to highlight how the steady-state airway and severely inflamed airway offer divergent environments for macrophage glycolytic metabolism. We describe how glycolytic metabolism in tissue resident alveolar macrophages (TR-AMs), interstitial macrophages (IMs), and monocyte-derived alveolar macrophages (Mo-AMs) relates to airway homeostasis and disease pathogenesis. We also provide an overview of experimental guidelines on how to assess metabolism at the cellular level using Seahorse-based bioenergetic analysis including a review of pharmacologic agents used to inhibit or activate glycolysis.

## 2 Airway microenvironment

The defining role of the lungs is to conduct gas exchange, and in doing so, oxygen is extracted from the external environment and into the bloodstream to drive metabolic processes at the cellular level throughout the body. This direct interface with the external environment necessitates cellular adaptation to the local microenvironment that is truly unique in relation to other specialized barrier sites. Under steady-state conditions, alveoli have one of the highest oxygen concentrations (PO_2 ~_100 mmHg at sea level, 13.2% O_2_) compared to other organs and compartments within the human body (1). Therefore, cells in the alveoli including TR-AMs are exposed to higher concentrations of O_2_ than most other cells in the body. In addition, luminal airway glucose concentrations are one-tenth of those observed in the blood, making the airway one of lowest glucose environments encountered by cells (2, 3). In contrast, the adjacent lung interstitium receives roughly 5.2% O_2_ (40 _mm_ Hg), and maintains glucose levels more comparable to those in blood (4). (**Table 1**) The high oxygen environment in the alveolar lumen is a direct consequence of the basic anatomy and physiology of the respiratory system. When coupled with the low glucose environment of the airway, high oxygen does not only deter growth of some pathogens but also shapes the metabolic preferences of specialized airway cells, particularly TR-AMs, which lack direct access to interstitial nutrients. For instance, epithelial cells in the airway express high levels of apical glucose transporters, which are thought to maintain low glucose concentrations in the airway for two key reasons (5). Rapid removal of glucose from the airway restricts nutrient availability to greatly limit opportunities for pathogen growth (2, 6). In addition, sodium-dependent glucose cotransporters in alveolar type II (ATII) cells serve as an important regulator of the airway surface fluid that is essential for normal respiratory mechanics (7). These cotransporters simultaneously encourage fluid reabsorption and glucose removal from the airway as a means to balance airway fluid and provide energy to fuel epithelial cell processes (5, 8, 9). ATII cells also contribute to the unique composition of the airway through the production of pulmonary surfactant, a complex mixture of proteins and lipids that principally serve to reduce alveolar surface tension and prevent lung collapse. Surfactant proteins also have several immunoregulatory properties, and the lipid-rich portion likely serves as an energy for cells within the airway, particularly TR-AMs, which have high fatty acid oxidation (FAO) gene expression (10, 11).

**Table 1:** Comparison of environmental and metabolic preferences amongst lung macrophage populations.

Macrophage	TR-AM	Mo-AM	IM
Environment			
**Steady-State Glucose**	0.4 ± 0.2 mM (Airway)	∼5.0 mM (Plasma)	Unknown
**Steady-State O_2_**	13.2% O_2_ (Airway)	1.5 - 4% O_2_ (Bone Marrow)	5.2% O_2_ (Lung Interstitium)
Metabolism			
**Steady-State**	OXPHOS (FAO)	Glycolysis OXPHOS	Glycolysis OXPHOS?
**Following stimulation with pathogen/LPS**	Unknown (Not Glycolysis)	Glycolysis	Glycolysis
Function			
**Steady-State**	- Phagocytosis - Maintain airway homeostasis	- N/A	- Immunoregulatory - Antigen presentation
**ARDS/Acute Inflammation**	- Phagocytosis - Regulated inflammatory response - Limit disease severity	- Pro-inflammatory - Enhance disease severity	- Both inflammatory and immunoregulatory - Can limit or enhance disease severity?

While the steady-state airway is marked by high oxygen levels, low glucose concentrations, and a lipid-rich environment, acute inflammation can significantly alter this composition. Viral pathogens, including influenza A and SARS-CoV-2, can cause severe lung injury and lead to acute respiratory distress syndrome (ARDS), which can lead to significant alterations to the airway microenvironment. ARDS is characterized by increased alveolar/endothelial permeability leading to alveolar fluid accumulation (12). Fluid accumulation results in increased airway glucose levels and impaired gas exchange creating local regions of high glucose and low oxygen, a near complete opposite environment compared to the steady-state lung (2, 13, 14). Moreover, ARDS dramatically reduces both the lipid and protein fractions of pulmonary surfactant within the airway to greatly alter homeostatic nutrient availability (15–18). Infiltrating immune cells also contribute to these alterations by sequestering oxygen and nutrients from resident cells (13).

Taken together, this information contextualizes the unique environment of the airway. At steady state, one would expect the high oxygen, low glucose, and lipid-rich airway environment to promote oxidative phosphorylation (OXPHOS) in resident airway cells. Under robust inflammation, the high glucose, low oxygen environment, and reduced lipid content would necessitate metabolic adaptation to anaerobic glycolysis to support resident airway cell effector function. These environmental features of the airway are critical in dictating resident cell effector function, and thus influence macrophage metabolism within the lung.

## 3 Tissue resident alveolar macrophages

TR-AMs serve as immune sentinels of the airway, where they must balance their maintenance roles of engulfing apoptotic cells, clearing cellular debris, and mitigating responses to benign antigens while also recognizing and mounting appropriate responses to pathogenic microorganisms (19). This often puts them at odds with traditional macrophage classification based on proinflammatory and anti-inflammatory polarization states (20). However, in general, TR-AMs are viewed to be less inflammatory than their monocytic counterparts and critical in regulating inflammation during ARDS and tissue repair following lung injury (21, 22). As mentioned in the previous section, the steady-state airway is an oxygen- and lipid-rich and glucose-limited environment. TR-AMs have evolved to become highly-specialized within this environment compared to their IM or Mo-AM counterparts. TR-AMs arise from fetal yolk monocytes and populate the airways during the first days of life (23, 24). Upon entry into the airway, TR-AMs undergo post-natal maturation where glycolysis is downregulated. Multiple groups have shown that preventing this downregulation of glycolysis through the deletion of Von Hippel-Lindau Protein (VHL), a key part of an E3 ubiquitin ligase complex responsible for targeting hypoxia-inducible factor 1-alpha (HIF-1α) for proteasomal degradation, produces immature TR-AMs (25, 26). VHL deletion leads to deficiencies in OXPHOS, lipid-handling, and self-renewal suggesting that downregulation of glycolysis is essential for normal TR-AM function at steady-state. It is thought that the airway environment provides cues for TR-AMs to downregulate HIF-1α target genes. This seems to be the case given that peritoneal macrophages and various macrophage precursors can be transferred into the airway and assume TR-AM phenotypical characteristics (27, 28).

The defining metabolic feature of TR-AMs is robust OXPHOS capacity. Deletion of mitochondrial transcription factor A (Tfam) reduces ETC complex expression and greatly reduces mitochondrial respiration across several immune cell subsets (29–31). When Tfam was deleted in macrophage populations across all cellular compartments in mice, TR-AMs experienced the largest decline in numbers (32). Tfam deletion resulted in impaired maturation, defective lipid handling, and apoptosis in TR-AMs, highlighting the importance of OXPHOS in TR-AM steady-state function (32, 33). We have previously shown that high dose ETC inhibition decreases TR-AM viability and that low dose ETC inhibition greatly reduces inflammatory capacity (34, 35). Collectively, these findings highlight the dominant role of OXPHOS in TR-AM steady-state metabolism.

Severe airway inflammation observed in ARDS leads to airway hypoxia and glucose influx. Bronchoscopic oxygen measurements in patients with cystic fibrosis harboring bacterial infections have recorded oxygen levels below 1%, and pimonidazole has been used to verify the presence of severely hypoxic alveoli during influenza infection in mice (36, 37). These conditions necessitate TR-AMs, which rely heavily on OXPHOS for maintenance function, to alter their metabolism in order to survive and aid in the resolution of severe inflammation. We and others have found that TR-AMs do not utilize glycolysis for proinflammatory processes *in vitro* (35, 38, 39). Moreover, we have shown that TR-AMs have comparatively low glycolytic protein expression and glycolysis alone cannot sustain normal TR-AM function in the presence of ETC inhibitors (34). This suggests that TR-AMs suffer from a glycolytic deficiency and cannot survive on glycolysis-derived energy production alone. However, when TR-AMs are exposed to hypoxia or pharmacological pseudohypoxia (inhibitors of prolyl hydroxylases), TR-AMs can assume a glycolytic phenotype that enhances TR-AM survival in the presence of ETC inhibitors (35). This hypoxic, glycolytic reprogramming is dependent on HIF-1α. We have shown that HIF-1α is not required for steady-state function or for proinflammatory processes in TR-AMs, but is critical in promoting TR-AM survival during influenza-induced ARDS in mice (35, 40). Significant TR-AM loss occurs during virus-induced (i.e., influenza, SARS-CoV-2) ARDS in humans and mice, and the degree of this loss is associated with poor outcomes (41–46). The exact mechanism of TR-AM depletion during ARDS remains unknown but evidence supports both cell-intrinsic and cell-extrinsic mechanisms of cell death. Gao et al. discovered that influenza infection decreases TFAM expression and causes mitochondrial damage in TR-AMs (33). After resolution of primary pneumonia in mice, surviving TR-AMs secrete higher levels of lactate in response to LPS (47). Mice treated intratracheally with HIF-1α activator (FG-4592) experienced reduced TR-AM loss, airway inflammation, and improved survival in a lethal model for influenza-induced ARDS (35). These findings suggest that TR-AM loss is linked to inadequate glycolytic adaptation under ARDS conditions that impair mitochondrial function (i.e. hypoxia and direct viral infection).

Given the well-established role of TR-AMs in ARDS, most of our knowledge on TR-AMs and their metabolism is based on data from clinical and experimental studies in ARDS. However, other lung diseases such as asthma or COPD may potentially affect or alter TR-AM metabolism and contribute to the pathogenesis of these chronic lung diseases. Others have suggested that glycolysis serves a role in TR-AM effector function outside of hypoxic adaptation. TR-AM glycolysis has been reported to be essential for interferon-driven cytokine production, IL-4 responsiveness, and allergen-induced inflammation (48–50). However, these studies rely heavily on 2-deoxy-D-glucose (2-DG) to inhibit glycolysis. 2-DG has extensive off-target effects, which are covered later in this review (See “Experimental Guidelines” section). Thus, while these studies are of value they should be interpreted with caution until genetic or other pharmacological approaches can confirm the results. Alveolar macrophages from smokers and patients with chronic obstructive pulmonary disease (COPD) exhibited impaired mitochondrial metabolism and increased glycolysis (51). Moreover, both monocyte-derived macrophages generated from blood and unsorted alveolar macrophages obtained via bronchoalveolar lavage from patients with COPD and treated *in vitro* exhibited alterations in mitochondrial reactive oxygen species generation (52, 53). However, one should be cautious about interpreting the results from these studies since alveolar macrophages are not sorted and the evaluation of metabolic profiles was performed on the whole alveolar macrophage population, which contains both TR-AMs and Mo-AMs; latter play a key driver role in the pathogenesis of COPD (54).Therefore, there is need for further studies in which alveolar macrophages are sorted to separate TR-AMs from Mo-AMs and other lung macrophages to determine whether COPD, asthma or other chronic lung diseases alter the metabolism of TR-AMs and other macrophages.

We have found that TR-AM metabolism and function is unperturbed by inhibiting glycolysis (34). We also observed no metabolic or functional changes when HIF-1α is deleted in TR-AMs, and that unlike other macrophage populations, LPS treatment does not result in HIF-1α translocation to the nucleus (40). In contrast, Zhu and colleagues have shown that *in vitro* treatment with Poly (I:C), a synthetic viral immunostimulant, or influenza infection induces HIF-1α in TR-AMs, but we were unable to reproduce these results (55, 56). In our hands, only hypoxia or pharmacological inhibition of prolyl hydroxylases induce TR-AM HIF-1α (34, 35, 40). Zhu and colleagues utilize GM-CSF in their TR-AM culture system. GM-CSF is critical in regulating TR-AM surfactant recycling *in vivo*, but it induces proliferation of TR-AMs in culture. Within the alveolus, TR-AMs form direct interactions with alveolar epithelial cells through extracellular receptors, like TGFBR and CD200R, that prevent aberrant proliferation at physiological concentrations of GM-CSF (19, 57). This could suggest that the observed, non-hypoxic HIF-1α induction in TR-AMs is associated with proliferation as opposed to inflammatory effector function. This will require new investigations examining the role of GM-CSF on HIF-1α induction in TR-AMs.

As with our work and those of others, it is important to recognize the limitations of most of these studies. Current knowledge on TR-AM metabolism, as well as most macrophage populations, is largely derived from experiments in isolated cells cultured in artificial media. These conditions do not mimic the local nutrient composition or O_2_ concentration and fail to account for cell-to-cell interactions (i.e., TR-AMs with AT II cells), which may directly or indirectly influence macrophage metabolism. Therefore, there is need for further studies that model not only the metabolic/O_2_ environment, but also take neighboring cells into account. Studies in cell metabolism have revolutionized our understanding of tumorigenesis. Recent investigations have incorporated spatial metabolomics integrated with spatial transcriptomics and proteomics in tumors to better understand macrophage metabolism and function (58, 59). These new approaches are likely to improve our understanding of TR-AM metabolism and function and lead to new areas of research.

In summary, under steady-state conditions, TR-AMs do express glycolytic machinery albeit at a much lower level than their counterparts of monocytic origin (i.e., recruited macrophages). Only hypoxia-mediated HIF-1α induction allows TR-AMs to perform functional glycolysis, which is defined as sustained lactate production to support cellular function in the presence of ETC inhibition. This supports the notion that TR-AM glycolysis evolved to support cellular function under hypoxic conditions and demonstrates that HIF-1α induction promotes TR-AM survival during ARDS, a condition marked by TR-AM loss and mitochondrial dysfunction. Future studies utilizing spatial omics will strengthen our knowledge and clarify discrepancies within the field of TR-AM metabolism in a context-specific manner.

## 4 Interstitial macrophages

Interstitial macrophages are resident macrophages that reside with the lung interstitium. Much less is known about IMs than TR-AMs, but IMs have been classified into two or three distinct cell populations based on such characteristics as cell surface markers, gene signatures, developmental origin, and location within the interstitium (60–63). The exact origin of IMs has been controversial. Most studies find that IMs are of monocytic origin, while others suggest that certain populations arise from the developmental yolk-sac, like TR-AMs (61, 63). However, it is mostly thought that yolk sac IMs are slowly replaced by monocytes in adult mice. IMs have been described as having more specialized roles based on whether they localize near sympathetic nerve bundles or alongside blood vessels, but in general most IM populations have been described as being immunoregulatory in that they secreted high levels of IL-10 and are well-equipped for antigen presentation (60, 62, 63). Interestingly, lung IMs share many of the same cell surface markers and gene signatures as macrophages found in the interstitium of the heart, skin, and gut suggesting that the lung IM phenotype is dictated by the generalized interstitial environment as opposed the specialized lung compartment (61).

As mentioned earlier, the lung interstitium environment is replete with glucose, but has much lower oxygen levels than the airway. This environment would likely dictate a more glycolytic phenotype for IMs compared to TR-AMs, which would be aligned with their developmental origins as monocytes. Under inflammatory conditions, IM expansion occurs and IMs can be readily found in the airway. It is still debated whether IM expansion occurs as a result of monocyte trafficking or self-renewal but it is likely a result from a combination of both mechanisms. Regardless, investigators have found that IMs favor glycolysis as their primary means for energy production. IMs secret higher lactate, have higher glycolytic gene expression, and lower FAO gene expression than TR-AMs (64). Mice infected with *Mycobacterium tuberculosis* and treated with 2-DG experienced IM loss and had a higher bacterial burden. This study found that IMs, proficient in glycolysis, were responsible for reduced bacterial burden, while TR-AMs, proficient in FAO, promoted bacterial growth (64). Collectively, IMs are glycolytically-oriented, which is beneficial in limiting the growth of *Mycobacterium tuberculosis*. The study of IM effector function and metabolism is still in its infancy, which leaves much to explore within the field.

## 5 Monocyte-derived alveolar macrophages

Monocyte-derived alveolar macrophages (Mo-AMs) are of monocytic origin, and enter the airway during acute inflammation where they both simultaneously retain monocytic characteristics while also being shaped into macrophages by the local airway microenvironment. Monocytes arise from adult hematopoietic stems cells (HSCs) within the bone marrow where oxygen concentrations range from 1.5 to 4% (65). HSCs rely on glycolysis for cellular maintenance, but harbor mitochondria that largely remain inactive as a means to limit reactive oxygen species damage in the hypoxic bone marrow (66–68). HSCs give rise to monocytes though colony stimulating factor 1 (CSF-1), and these monocytes circulate in the blood with an average lifespan of 1-3 days. Glucose transporter-1 (GLUT-1)-dependent glucose metabolism maintains monocyte homeostasis and migratory capacity to the extent that serum glucose concentrations correlate with monocyte numbers (69, 70). Representative of monocyte-derived macrophages, murine bone marrow-derived macrophages (BMDMs) have been used extensively for *in vitro* immunometabolism studies. These studies have found that robust glycolysis is a critical mediator of proinflammatory responses in BMDMs (71–75).

Under diseased conditions, the affected tissue generates chemokine gradients that recruit monocytes to the origin of the inflammatory insult. In this case, both general macrophage differentiation pathways are activated as well as tissue-specific pathways that give rise to a more specialized recruited macrophage. For example, recruited macrophages existing in the tumor microenvironment share generalized macrophage characteristics with recruited Mo-AMs, but will also possess unique characters more aptly tailored to the specific tissue site (76, 77).

By the time Mo-AMs have constituted the airway in ARDS, the low glucose/high oxygen/lipid-rich environment of the steady-state lung has dramatically shifted. From a nutrient perspective, the inflamed airway more closely resembles the high glucose/low oxygen niche of the bone-marrow from which the Mo-AMs arise. These conditions, along with monocytic imprinting, favor a functional glycolytic phenotype to fuel cellular processes. In the case of viral infection, it is well accepted that Mo-AMs exacerbate lung injury (42, 46, 78–81). It may be that Mo-AMs are initially well-suited to limit viral replication or to later limit secondary bacterial infection upon recovery from viral infection, but their presence is associated with excessive inflammation and poor outcomes (82, 83). Our group has observed that Mo-AMs express high levels of glycolytic and proinflammatory genes and low levels of OXPHOS-related genes in a murine model of influenza-induced ARDS (34, 35). This Mo-AM gene expression pattern remains relatively stable across the acute phase of the infection time course, while TR-AMs, if they do not experience cell death, shift from an OXPHOS dominant gene profile to a glycolysis-dominant gene profile as ARDS progresses (35). Work by Li et al. confirms these findings where Mo-AMs have high glycolysis and low OXPHOS during influenza infection (81). Mo-AMs recovered from patients with severe SARS-CoV-2 infection exhibited high HIF-1α expression and enrichment of hypoxia genes while also exhibiting reduced enrichment of OXPHOS genes, suggesting that Mo-AM metabolism is conserved in viral-induced ARDS (56).

Following successful resolution of viral infection, both self-renewing TR-AMs and Mo-AMs reconstitute the alveolar niche in response to viral-induced TR-AM death. Several studies have found that these persisting Mo-AMs are significant contributors to pulmonary fibrosis (84–90). In murine models of bleomycin-induced pulmonary fibrosis, TR-AM numbers decrease during the inflammatory phase and are almost entirely replaced by Mo-AMs (85, 91). Misharin and colleagues demonstrated that murine lung injury with either influenza or bleomycin resulted in 50% of the alveolar niche being composed of Mo-AMs one year post injury (85). These long-lived Mo-AMs may contribute to collagen deposition through enhanced secretion of profibrotic factors, including TGF-β (90). Additionally, murine Mo-AMs remain glycolytic following injury and the development of fibrosis (86). Analysis of human lungs has also identified associations between Mo-AMs and profibrotic lesions, and airway macrophages from patients with idiopathic pulmonary fibrosis express higher levels of GLUT-1 compared to healthy controls (88, 92–94).

Other groups have identified long-lived Mo-AMs to be beneficial. Aegerter et al. showed that Mo-AMs residing in the lung following influenza challenge secret more IL-6 than their TR-AM counterparts making them more effective in limiting secondary bacterial infection (82). Another group found that Mo-AMs constituted the airway following gamma herpesvirus infection and that these cells conferred protection against allergic asthma (95).

Collectively, glycolytically oriented Mo-AMs repopulate the airway following airway injury and inflammation. These cells have been associated with poor outcomes in ARDS and have identified in driving pulmonary fibrosis but may be beneficial in limiting secondary bacterial infection and allergic responses. More studies are needed to understand the appropriate balance of Mo-AMs and TR-AMs to not only prevent the development of chronic lung disease, but to also bolster the beneficial functions of Mo-AMs.

## 6 Experimental guidelines

Given the unique metabolic characteristics exhibited by TR-AMs compared to other macrophage populations within the body, it is worthwhile to discuss best experimental practices when assessing the glycolytic phenotype of lung macrophages. In this section, we will discuss the existing TR-AM culture systems including their metabolic implications and review experimental reagent selection for inhibiting or activating glycolysis and the interpretation of bioenergetics data. We will also provide information about the emerging new techniques that enable evaluation of metabolic profiles at single cell level. Ensuring that these best practices are followed will allow for the most accurate interpretation of macrophage metabolic phenotype and promote rigorous data reproducibility.

### 6.1 TR-AM culture systems

Sample collection is a major limitation to studying TR-AMs. A single mouse will yield approximately 300,000-500,000 TR-AMs via bronchoalveolar lavage, and obtaining human TR-AM specimens has several barriers making the endeavor impractical for routine use. Moreover, *in vitro* TR-AMs maintain high expression of many core TR-AM-specific genes, but genes related to the expression of key TR-AM functions, including surfactant handling and lipid metabolism, decrease with extended time in culture. This likely reflects the loss of cues from alveolar niche, and highlights the challenges of studying TR-AM metabolism *in vitro* (96). Granulocyte-macrophage colony-stimulating factor (GM-CSF), transforming growth factor β receptor (TGF-βR), and peroxisome proliferator-activated receptor gamma (PPARγ) have all been identified as critical regulators of TR-AM maturation and homeostasis (11, 23, 97). Gorki et al. established a system where isolated TR-AMs were cultured in the presence of GM-CSF, TGF-β, and rosiglitazone (PPARγ activator) (98). This combination yielded TR-AMs that could be expanded for months while maintaining functional and morphologic features found in freshly isolated TR-AMs. Another group took human monocytes and transformed them into “TR-AM-like cells” using bovine surfactant, GM-CSF, TGF-β, and IL-10 (99). Like the murine culture system, these human “TR-AM-like cells” exhibited a morphological, transcriptional, and metabolic profile more similar to freshly isolated human TR-AMs than to human monocyte-derived macrophages.

The full extent of how these different culture systems recreate functional TR-AM metabolism is debatable and necessitates further studies. In isolation, GM-CSF has been shown to be critical in regulating mitochondrial function in both TR-AMs and BMDMs (100, 101). GM-CSF has also been shown to enhance inflammation and glycolysis in murine BMDMs (102). TGF-β signaling regulates lipid metabolism in TR-AMs but was found to enhance glycolysis and reduce proinflammatory cytokine production in murine peritoneal macrophages (97, 103). Trying to recreate the lung niche through different cytokines and nutrients is an important endeavor. Providing TR-AMs with lipids through surfactant supplementation goes a step beyond adding lung specific cytokines and growth factors (99). However, to truly capture the alveolar niche, we may have to go a step further and consider co-culturing TR-AMs with alveolar epithelial cells. As mentioned before, TR-AMs form direct interactions with alveolar epithelial cells through extracellular receptors, like TGFBR and CD200R. Under these circumstances, TR-AMs do not proliferate at physiological concentrations of GM-CSF, and TGF-β serves as an inhibitor of macrophage activation. Thus, it is likely that GM-CSF and TGF-β may both keep TR-AMs more “AM-like” in culture, while also promoting a TR-AM phenotype that would not be observed within the context of the alveolar environment. Nevertheless, long-term TR-AM culture systems should be utilized with continual modifications being made in an attempt to make it easier and more physiologically relevant to study TR-AM function *in vitro*.

### 6.2 Agents used to inhibit or activate glycolysis

Inhibiting or activating glycolysis allows one to assess the full functional relevance of glucose catabolism in a given cell type. The most commonly used reagent for inhibiting glycolysis is 2-deoxy-D-glucose (2-DG), a stable glucose analog that is taken up by glucose transporters and retained in the cell following phosphorylation by hexokinase. 2-DG accumulation competitively inhibits the conversion of glucose to glucose-6-phoasphate leading to glycolytic arrest. While this is the desired outcome in assessing the effects of glycolysis inhibition on cellular function, 2-DG has been identified to have off several off target effects (104). Mainly, 2-DG inhibits N-linked glycosylation of proteins by also serving as a mannose mimic. N-linked glycosylation inhibition prevents proper protein folding and promotes protein retention in the endoplasmic reticulum (ER) leading to activation of the unfolded protein response and subsequent apoptosis (105). 2-DG-induced ER stress has been shown to inhibit both inflammatory capacity through COX-2 and fatty acid synthesis independently of glycolysis inhibition (106–108). Most importantly, commonly used 2-DG concentrations (10 mM) have been shown to inhibit oxidative phosphorylation independently of glycolysis in macrophages within one hour of treatment (109). These off target effects render 2-DG a less than optimal tool for studying the reliance of macrophages on glycolysis at steady-state or for effector functions. Alternatively, one could use oxamate, a competitive inhibitor of lactate dehydrogenase, that not only inhibits lactate production, but also inhibits overall glycolytic activity (110, 111). Manipulating culture media conditions to greatly reduce glucose availability via low glucose concentrations or by replacing glucose with galactose to greatly inhibit the rate of glycolysis, serve as valuable tools to study macrophages reliance on glycolysis without compromising cellular glycosylation (109). We have shown that overnight treatment of bone marrow-derived macrophages with 2-DG, but not oxamate or glucose-free media [non-dialyzed fetal bovine serum media supplementation provides roughly 0.4-0.8mM glucose (112)], induces cleaved caspase 3 expression, signifying a commitment to apoptosis (34, 113). Thus, we would suggest that 2-DG be avoided in studying the role of glycolysis in cellular effector function, and for 2-DG-based studies to be interpreted in such a way as to not overestimate the role of glycolysis in shaping metabolic phenotype.

Glycolysis can also be induced through the HIF-1α activation, which provides a method for assessing the full range of glycolytic phenotype in lung macrophages. This can be done through prolyl hydroxylase inhibition using hypoxia or pharmacological inhibitors. Under normal oxygen conditions, oxygen-dependent prolyl hydroxylases mark HIF-1α for proteasomal degradation. As oxygen levels decrease, prolyl hydroxylase activity becomes reduced so that HIF-1α accumulates, then enters the nucleus, and activates the hypoxic transcriptional response resulting in the upregulation of glycolytic enzyme expression (114). Hypoxia may alter macrophage inflammatory capacity independently of HIF-1α induction or changes in glycolysis so pharmacological inhibitors may offer a cleaner approach to studying how upregulation of glycolytic metabolism alters cellular function (35, 115). The most commonly used pharmacological prolyl hydroxylase inhibitor is dimethyloxalylglycine (DMOG), a synthetic analogue of α-ketoglutarate. This is a great tool for assessing HIF-1α induction and glycolytic phenotype, but it can also directly inhibit mitochondrial function and significantly reduce macrophage inflammatory capacity (34, 116–118). Thus, we suggest using second generation pharmacological prolyl hydroxylase inhibitors such as Roxadustat (FG-4592). We have found that Roxadustat can stabilize HIF-1α and significantly enhance glycolysis without significantly altering macrophage inflammatory capacity (35, 40). Roxadustat mildly suppress mitochondrial function in macrophages, but this effect is lost in HIF-1α knockout cells demonstrating that decreased respiration is linked to canonical HIF-1α activation (40). In contrast, decreases in cellular respiration following DMOG treatment precede HIF-1α activation and are independent of HIF-1α signaling, highlighting that DMOG has unspecified effects on cell metabolism (119).

### 6.3 Seahorse-based assessment of glycolysis

Seahorse (Agilent) metabolic flux assays have enabled users to assess the overall metabolic fitness of a given cell type through the measurement of the oxygen consumption rate (OCR) and extracellular acidification rate (ECAR). Mitochondrial oxygen consumption can be determined as the total OCR minus the OCR that remains after exposure to specific electron transport chain (ETC) inhibitors (120, 121). Thus, the OCR of a given cell type serves as a direct readout for the mitochondrial electron transport rate. The ECAR is routinely used as a direct surrogate in the measurement for glycolytic metabolism, but the ECAR requires a more nuanced analysis to determine whether acidification is derived from glycolysis. The conversion of one glucose molecule to two lactate molecules results in the net release of two protons that acidify the media, however, other metabolic reactions can also contribute to the ECAR. CO_2_ generated via the TCA cycle is readily converted to carbonic acid which can contribute media acidification (122). In fact, the complete oxidation of one glucose molecule by the TCA cycle yields six protons that can contribute to media acidification. This suggests that the ECAR alone can be an unreliable measure of cellular glycolysis, and we have found this to be the case in studying TR-AMs.

**Figure 1:**
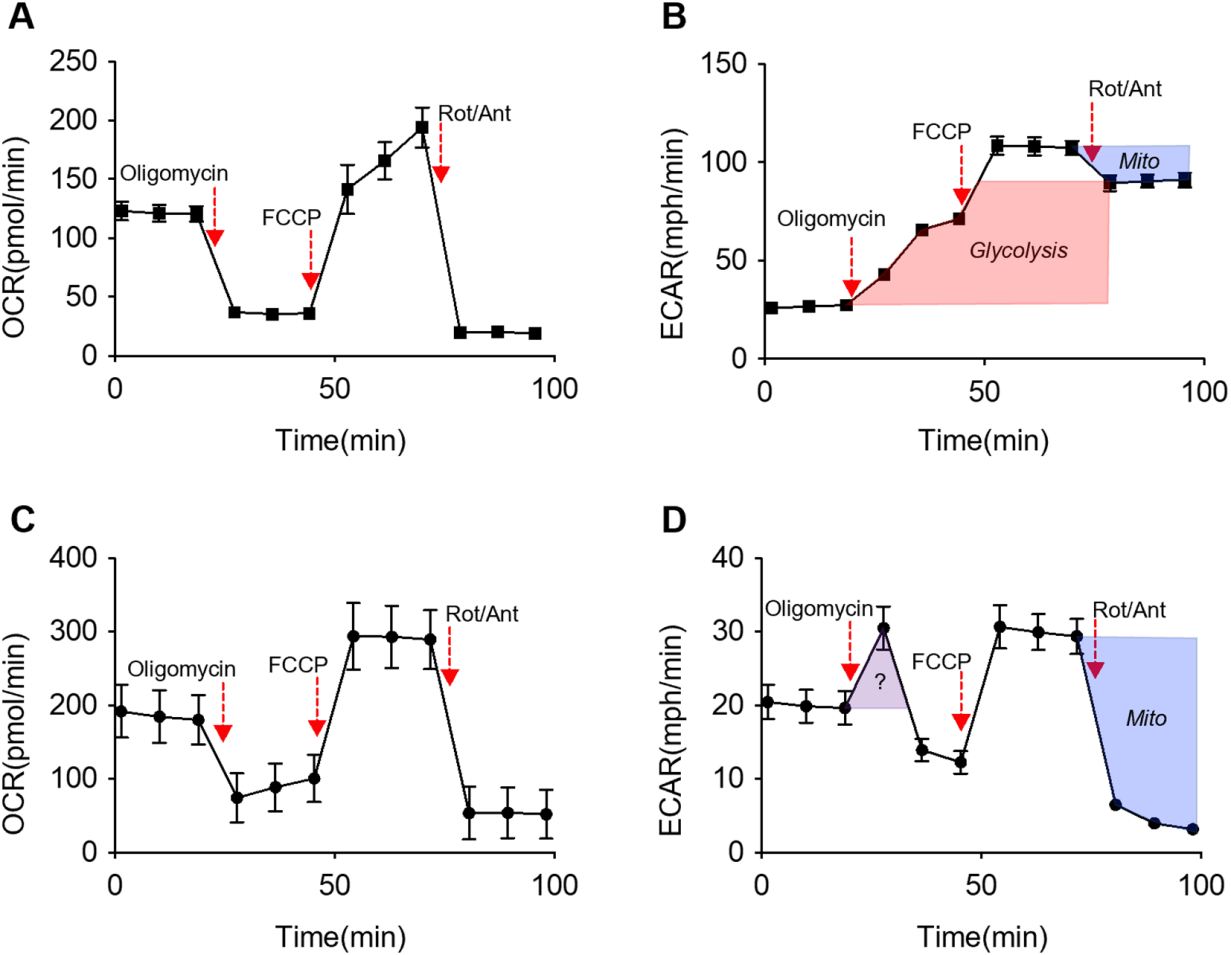
Analysis of reciprocal ECAR measurements during mitochondrial stress test to assess glycolytic capabilities in response to mitochondrial inhibition. **(A, B)** BMDMs and **(C-D)** TR-AMs were treated sequentially with oligomycin (ATP synthase inhibitor), FCCP (uncoupler) and Rotenone/Antimycin A (complex I and III inhibitor, respectively). Red shading denotes glycolytic contribution to ECAR. Blue shading denotes Mitochondrial (Mito) contribution to ECAR. Purple shading denotes unknown contribution to ECAR.

The standard Seahorse assay for assessing glycolytic metabolism is the glycolysis stress test. While this test is essential in measuring macrophages responsiveness to glucose, it often fails to assess the cells’ overall reliance on glycolysis for essential metabolic activity. For this reason, we would also suggest a thorough interrogation of the ECAR during a Mitochondrial Stress Test to get a complete understanding of the metabolic phenotype (**Figure 1**). One can determine cell’s reliance on glycolysis by assessing the ECAR changes in the presence of mitochondrial inhibitors. If a cell is truly glycolytic, then the ECAR rate should increase, and remain at a sustained, higher rate in the presence of oligomycin. The ECAR will also increase following the decoupler (FCCP), and this increase is a result of CO_2_-derived carbonic acid from the TCA cycle. Lastly, the ECAR of a cell that exhibits true glycolytic capabilities will remain relatively unresponsive to complex I and III inhibitors (Rotenone and Antimycin) suggesting that the majority of acidification is derived by glycolysis. This is quite visible in BMDMs (**Figure 1B**). In the case of TR-AMs, a higher, sustained ECAR is only observed following FCCP injection, and then the ECAR immediately returns to below baseline rates following Rotenone/Antimycin injection demonstrating that the contribution to the ECAR is entirely mitochondria-derived carbonic acid (**Figure 1D**). The spike in TR-AM ECAR in response to oligomycin, and its subsequent and immediate collapse is harder to parse out, but it does suggest that TR-AMs cannot sustain metabolic activity through anaerobic glycolysis. Others have documented the spike and crash in TR-AM ECAR following oligomycin injection (50, 81, 123).

Seahorse is a valuable tool in evaluating cellular metabolism *ex vivo* but is more limited in assessing cellular metabolism of rare and heterogeneous cell populations. New approaches like single-cell energetic metabolism by profiling translation inhibition (SCENITH) aim to circumvent these limitations (124). SCENITH relies on the knowledge that a substantial amount of the total energy produced through metabolic pathways is immediately consumed by protein synthesis machinery so that ATP production and protein synthesis are tightly coupled (125, 126). Measuring protein synthesis (puromycin incorporation) via fluorescence-activated cell sorting (FACS) in the presence of metabolic inhibitors can be used for metabolic profiling (124). More importantly, this methodology could provide single cell resolution of energy metabolism from heterogenous blood and tumor samples. This method is not without caveats in that the processing of tissue and FACS can lead to alterations in cellular metabolism (127). Likewise, selecting metabolic inhibitors with off-target effects could lead to inaccurate data interpretation. This is why the best approach for understanding cellular metabolism is to utilize multiple technologies. An “integrated toolbox to profile macrophage immunometabolism” that utilizes functional assays, Seahorse analysis, metabolic dyes, metabolomics, and SCENITH has been proposed to more conclusively determine the metabolic characteristics of a cell (128). Each methodology alone has its limitations, which can be counteracted through a multifaceted approach. We hope that this experimental guideline section can provide researchers with the basic tools for generating the most accurate and rigorous data in lung macrophage metabolism.

## 7 Conclusion

Despite recent advances in our understanding of pulmonary macrophage metabolism, key questions remain. Gene expression data suggest that TR-AMs utilize FAO at steady-state, but no one has mechanistically identified their preferred metabolic fuel through functional assays. Moreover, outside of developmental and environmental cues, we do not fully understand why TR-AMs cannot sustain cellular processes through glycolysis. Gaining a better understanding of the dynamics of TR-AMs, Mo-AMs, and IMs in disease inflammation and resolution may provide clues to some of these questions and also aid in the development of therapeutics to reduce mortality from ARDS and pulmonary fibrosis.
